# Supplementary material for: Learning curve and surgical outcome of robotic assisted colorectal surgery with ERAS program
Source: Sci Rep. 2022 Nov 29;12:20566. doi: 10.1038/s41598-022-24665-w (PMC9709162; doi:10.1038/s41598-022-24665-w)
Supplement: Supplementary file 1 — Supplementary Information 1. [file 41598_2022_24665_MOESM1_ESM.zip › LSC Raw data-Ñ[▒K/138/H_ñw╝╨░O▒Kñσ.pdf]

正式摘要(電子簽章：00000022619875[illegible])

W92 / W92 ☐ 英文標題(注意:無英文姓名-請補病人基本檔!) ☐【讀取】憑證

臺中榮民總醫院

出院病歷摘要

轉入號： 轉出號：

[redacted]

**(8)**流水編號	
**(9)**入院日期	110/09/22 (2021) 直腸外科 W92 - 03
**(10)**轉科日期	
**(11)**出院日期	110/09/29 (2021) 住院天數計 7 日

診斷

- 入院 :1. Rectal adenocarcinoma, cT3N2aM0, stage IIIB
  -s/p neoadjuvant CCRT with IMAT 50 Gy/25 fr. to rectum (2021/06/16-07/20) and Ufur 2# BID
- 出院 :1. Rectal adenocarcinoma, cT3N2aM0, stage IIIB
  -s/p neoadjuvant CCRT with IMAT 50 Gy/25 fr. to rectum (2021/06/16-07/20) and Ufur 2# BID
  -s/p laparoscopic low anterior resection and ileostomy on 2021/09/23

主訴

- anal pain and bleeding after defecation for 2 months

病史

- This 72-year-old man denid systemic disease was suffered from anal pain and bleeding after defecation for 2 months, he also mentioned body weight loss 5kg in 2 months and felt easily dizziness and malaise. He had visited LMD and colonoscopy survery (2021/06/05) showed locally advanced rectal tumor, thus he was referred to our OPD. Abdominal CT(2021/06/08) revealed rectum cancer with suspicious perirectal invasion and regional lymphadenopathy, cT3N2a, stage IIIB. We arranged neoadjuvant CCRT with IMAT 50 Gy/25 fr. to rectum (2021/06/16-07/20) and Ufur 2# BID.
  This time, he was admitted for laparoscopic low anterior resection.

體檢發現

-
  - General appearance :
    - easy looking , conscious clear , without cardiopulmonary distress
    - Vital signs自動帶入date 體溫(BT) 脈搏(P) 呼吸(R) 血壓(BP) 身高(BH) 體重(BW) BMI
      20210922 36.7℃ 80-次/分 18-次/分 118/72mmHg 171cm 53.9kg 18.4
    - Vital signs: B.P. 118/72 mmHg , P.R. 80 /min , R.R. 18 /min , B.T. 36 ℃
  - HEENT :
    - Eyes: no anemic conjunctiva, no icteric sclera, normal EOM
    - ENT: no otorrhea, no rhinorrhea, no erythematous mucosa, no injected tonsil, no deformity
  - Neck: supple, freely movable, trachea midline,no deformity, no engorged jugular vein , no palpable thyroid or lymph node enlargement
  - Chest:
    - Inspection: Grossly normal, symmetric and fully expansion, no accessory muscle used, no operation scar, no spider angiomata, no gynecomastia
    - Palpation: no palpable mass or nodule, no subcutaneous emphysema, no tenderness
    - Ausculation: clear breathing sound
  - Heart:
    - Percussion: normal heart size
    - Ausculation: regular heart beat, normal S1, S2, no gallop, no murmur
  - Abdomen:
    - Inspection: in
      - flat contour, symetrically, no scar, no stria, no dilated veins, no rashes
    - Ausculation: normoactive bowel sound, no abdominal bruits
    - palpation: soft, no muscle guarding or rigidity, no local tenderness, no rebounding pain, no palpable mass, no palpable pulsation
    - Percussion: no shifting dulllness, no hepatomegaly, no splenomagaly
  -

Peripheral pulsation :	CA	BA	RA	FA	PA	PTA	DPA
Right side :	++	++	++	++	++	++	++/+++
Left side :	++	++	++	++	++	++	++/+++

  - Genito-anus: no tenderness or palpable mass lesion or rectal shelf, normal external genitalia, normal anal tone, brown stool

CRS CONDITION

  - CEA: 3.66 ng/ml
  - Imaging studies(Sono , CT of abdomen and chest Films):
    - 檢驗項目 :CT ABDOMEN-SURVEY 報告日期 :1100611
      主 訴 :
      Result:Y Finding: Imaging Report Form for Colorectal Carcinoma (AJCC 8th) 1. Imaging Date: 20210608 Imaging Modality, Imaging by CT scan Imaging Protocol: Post IV contrast CT scan of the whole abdomen with 600cc oral contrast. 2. Tumor location / Size Location: Rectum Size: Measurable: 4.4 cm (largest diameter) 3. Tumor invasion Tumor invades non-peritonealized pericolonic or perirectal tissues 4. Regional nodal metastasis N2 | N2a Four to six regional lymph nodes are positive. 5. Distant metastasis (In this study) No or Equivocal 6. Other findings: > Segmental asymmetric wall thickening of rectum about 4.4cm in length (Cor:27-32), may compatible with rectum cancer. Slight fat stranding at perirectal region, perirectal invasion cannot be ruled out. > Several small lymph nodes at perirectal region, regional lymphadenopathy should be considered. > Multiple low attenuation nodules over right lobe of liver, r/i hepatic cysts, suggest follow up > Multiple renal cysts over bilateral kidneys, up to 0.8cm > Prominent of prostate gland with calcifications. Suggest check up PSA. > Bil. hydroceles. > The pancreas, spleen, gallbladder, and visible lung fields are without specific abnormality > No other significant lymphadenopathy at retroperitoneum and bil. pelvic region > No osteolytic or osteoblastic bone lesion is identified in visible bony structure.
      Impression: 1. R/I rectum cancer with suspicious perirectal invasion and regional lymphadenopathy.
      2. For other details, please see the above descriptions.
  -
    - Lung

手術日期及方法

- 手術日期及方法20210923 Low anterior resection with colo-anal anastomosis Ileostomy Laparoscopy
- 手術結果及發現 :
  - 1. An proturded tumor, about 2cm x 3 cm in size, located at middle rectum, didn't penetrate through the serosa. No palpable lymph nodes at mesocolon, along intermediate vessel to IMA root. 2. No visible liver metastasis or peritoneal seeding

住院治療經過

- After admission, preoperative ERAS care was done, he underwent laparoscopic low anterior resection and ileostomy smoothly on 2021/09/23. After surgery, he had regained conscious and tolerated well under PCA control. Postoperative ERAS care was applied. He had semi-liquid oral diet smoothly at POD-2. Penrose drainage tube was removed at POD-5. He was discharged.

合併症

- Nil

一般檢查

- 累積報告 :| | | | | | | | | | | | | | | |

-

DATE	TIME	ALB	TP	BIL.T	BIL.D	ALKP	AST	ALT	LDH	AMY	LIPASE	NH3	Ferritin	UA
1100922	1636	3.8		0.67	0.18		36	38						
1100923	1833	3.4		1.05	0.25		40	36						

DATE	TIME	WBC	RBC	HGB	HCT	MCV	PLT	BLST	NEUT	BAND	SEG	LYM	MON	EOS	BAS
1100922	1629	4260	2.51	10.2	28.6	113.9	141		72.4			15.5	10.3	1.6	0.2
1100923	1831	7770	2.55	10.0	28.4	111.4	128		90.8			6.9	2.3	0.0	0.0

DATE	TIME	NA	K	CL	BUN	CREAT	eGFR	CA	Mg	P	CRP	ESR	PCT	D-Dimer	LACTATE
1100922	1636	139	3.9	106	22	0.97		8.4							
1100923	1832														15.6
1100923	1833	139	3.6		15	0.90	88.16	8.1							

DATE	TIME	GLUCOSE	HbA1C	CHOL	TG	HDL	LDL	KETONE	CK	CKMB	TRO-T	PT-P	APTT-P	NT-pro BNP
1100922	1634		5.3											

DATE	TIME	PH	PCO2	PO2	SO2	BEB	HCO3	TCO2	HGB	FIO2
1100923	1830	7.420	37.4	165.0	99.5	-0.4	23.7	24.9		

- 放射線報告 :檢驗項目 **:CHEST AP VIEW (PORTABLE)** 報告日期 **:1100923**
  主 訴：
  Result:N
  Finding:
  > Borderline heart size.
  > Tortuosity and atherosclerotic changes of T-aorta.
  > Slightly increasing interstitial infiltration over bilateral lung fields.
  > DJD changes with marginal spur formation of T-L spine.
  > Increased opacity over upper mediastinum, nature to be determined, suggest clinical correlation.
  > EKG leads placement.
  Impression:
  As above description.

特殊檢查

病理報告

其它

出院時情況

- 3.改門診治療

出院指示

-

藥名	劑量	劑量單位	用法	頻次	數量	數量單位	說明	天數	單位
(□)Takepron OD tab 30mg	1	TAB	PO	QDAC	7	TAB	TH	7	天
Otilonium tab 40mg	1	TAB	PO	BID	14	TAB	TH	7	天
Ultracet tab	1	TAB	PO	Q6H	28	TAB	TH	7	天
Urief tab 4 mg	1	TAB	PO	BID	14	TAB	TH	7	天

出院計劃

- 門診日期
  - date on 2021/10/05

主治醫師_2062F 蔣鋒帆_____ / 住院醫師_2336L 洪凌嚴_____

正式摘要(電子簽章：**000000226198756)**

W92 / W92

doctorRank:B
